# Drinking-Water Disinfection By-products and Semen Quality: A Cross-Sectional Study in China

**DOI:** 10.1289/ehp.1307067

**Published:** 2014-04-04

**Authors:** Qiang Zeng, Yi-Xin Wang, Shao-Hua Xie, Liang Xu, Yong-Zhe Chen, Min Li, Jing Yue, Yu-Feng Li, Ai-Lin Liu, Wen-Qing Lu

**Affiliations:** 1 Department of Occupational and Environmental Health, School of Public Health, Tongji Medical College, Huazhong University of Science and Technology, Wuhan, Hubei, PR China; 2 Key Laboratory of Environment and Health, Ministry of Education & Ministry of Environmental Protection, and; 3 State Key Laboratory of Environmental Health (Incubating), School of Public Health, Tongji Medical College, Huazhong University of Science and Technology, Wuhan, Hubei, PR China; 4 School of Public Health and Primary Care, The Chinese University of Hong Kong, Hong Kong SAR, PR China; 5 Reproductive Medicine Center, Tongji Hospital, Tongji Medical College, Huazhong University of Science and Technology, Wuhan, Hubei, PR China

## Abstract

Background: Exposure to disinfection by-products (DBPs) has been demonstrated to impair male reproductive health in animals, but human evidence is limited and inconsistent.

Objective: We examined the association between exposure to drinking-water DBPs and semen quality in a Chinese population.

Methods: We recruited 2,009 men seeking semen analysis from the Reproductive Center of Tongji Hospital in Wuhan, China, between April 2011 and May 2012. Each man provided a semen sample and a urine sample. Semen samples were analyzed for sperm concentration, sperm motility, and sperm count. As a biomarker of exposure to drinking-water DBPs, trichloroacetic acid (TCAA) was measured in the urine samples.

Results: The mean (median) urinary TCAA concentration was 9.58 (7.97) μg/L (interquartile range, 6.01–10.96 μg/L). Compared with men with urine TCAA in the lowest quartile, increased adjusted odds ratios (ORs) were estimated for below-reference sperm concentration in men with TCAA in the second and fourth quartiles (OR = 1.79; 95% CI: 1.19, 2.69 and OR = 1.51; 95% CI: 0.98, 2.31, respectively), for below-reference sperm motility in men with TCAA in the second and third quartiles (OR = 1.46; 95% CI: 1.12, 1.90 and OR = 1.30; 95% CI: 1.00, 1.70, respectively), and for below-reference sperm count in men with TCAA in the second quartile (OR 1.62; 95% CI: 1.04, 2.55). Nonmonotonic associations with TCAA quartiles were also estimated for semen parameters modeled as continuous outcomes, although significant negative associations were estimated for all quartiles above the reference level for sperm motility.

Conclusion: Our findings suggest that exposure to drinking-water DBPs may contribute to decreased semen quality in humans.

Citation: Zeng Q, Wang YX, Xie SH, Xu L, Chen YZ, Li M, Yue J, Li YF, Liu AL, Lu WQ. 2014. Drinking-water disinfection by-products and semen quality: a cross-sectional study in China. Environ Health Perspect 122:741–746; http://dx.doi.org/10.1289/ehp.1307067

## Introduction

Several studies have reported a worldwide decline in semen quality in the general population over the past few decades (Auger et al. 1995; Carlsen et al. 1992; Irvine et al. 1996; Jorgensen et al. 2001; Li et al. 2009). These findings have led to considerable interest and debate about whether exposure to certain environmental chemicals, especially reproductive toxicants, contributes to declining semen quality (Sharpe and Irvine 2004). Disinfection by-products (DBPs) are a group of environmental chemicals formed during the process of chlorinating drinking water; this process is used extensively worldwide, including in China, to reduce the incidence of waterborne diseases. Since the 1970s, when DBPs were first reported, the potential adverse health effects of DBPs have been an increasing concern.

To date, > 600 types of DBPs with different physicochemical and toxic properties have been identified in chlorinated drinking water (Richardson et al. 2007). The ubiquity of DBPs in the domestic water supply leads to daily and long-term human exposure through various routine water-use activities (e.g., drinking, bathing, showering, and swimming) (Nieuwenhuijsen et al. 2009). Consequently, based on the detection of trichloroacetic acid (TCAA) in urine, a biomarker that reflects ingestion of DBPs in chlorinated drinking water, exposure to DBPs has been reported in > 75% of a representative sample of the U.S. general population (Calafat et al. 2003).

Toxicological studies have reported that exposure to DBPs—especially the two most abundant groups of DBPs, trihalomethanes (THMs) and haloacetic acids (HAAs)—adversely affects male reproductive health in rats. Exposure to THMs has been found to reduce serum testosterone and sperm motility, as assessed by significantly decreased mean sperm straight-line, average path, and curvilinear velocities (Klinefelter et al. 1995; Potter et al. 1996). Oral exposure to HAAs has consistently been observed to acutely affect spermatogenesis, distort sperm motility and morphology, and impair male reproductive competence in rats (Linder et al. 1994a, 1995, 1997a, 1997b). Furthermore, recent studies in rats and rabbits have reported that exposure to DBPs is associated with significantly decreased levels of SP22, a sperm membrane protein that is highly correlated with male fertility (Klinefelter et al. 2002, 2004; Veeramachaneni et al. 2007).

The accumulating evidence from toxicological studies suggests that exposure to drinking-water DBPs may pose a threat to male reproductive health in humans. However, only a limited number of epidemiological studies to date have reported an association between exposure to drinking-water DBPs and semen quality, with inconsistent results. Previous studies used DBP concentrations in water distribution systems as surrogates of exposure, which may result in misclassification of exposure and bias the observed associations (Fenster et al. 2003; Luben et al. 2007). In recent studies, we used DBP biomarkers to improve the assessment of exposure and found a potential relationship between DBP exposure and decreased semen quality (Xie et al. 2011; Zeng et al. 2013). However, limited sample sizes in our previous studies have often been insufficient to produce precise results (Nieuwenhuijsen et al. 2009). Consequently, the effect of exposure to drinking-water DBPs on semen quality in humans remains uncertain.

Therefore, we conducted a large-scale study to examine the relationship between DBP exposure and semen quality in a Chinese population. We classified DBP exposure using urinary TCAA concentration as a biomarker; TCAA has been reported to be a valid biomarker of DBP ingestion through chlorinated drinking water (Calafat et al. 2003; Froese et al. 2002; Kim et al. 1999; Zhang et al. 2009b).

## Methods

*Study design and participants*. We designed a hospital-based cross-sectional study to examine the relationship between exposure to drinking-water DBPs and semen quality in a Chinese population. We recruited study participants from men who came to the Reproductive Center of Tongjing Hospital in Wuhan, China, to seek semen analysis. We conducted this study in two phases. A total of 1,278 men in the first phase (April through July 2011) and 1,262 men in the second phase (March through May 2012) agreed to participate in the study. The number who agreed relative to the number recruited was the same for men with and without fertility problems. The study was approved by the Ethics Committee of Tongji Medical College, and informed consent was provided by each participant at enrollment.

Because some chemicals, such as trichloroethylene (TCE), 1,1,1-trichloroethane (TRI), and perchloroethylene (PERC), can be metabolized into TCAA and may result in the misclassification of exposure to DBPs [Agency for Toxic Substances and Disease Registry (ATSDR) 1995, 1996, 1997], we excluded 81 men who reported occupational exposures to synthetic materials such as glues, paints, and lubricants that might be a source of exposure to these chemicals. We also excluded 332 azoospermic men because the mechanism responsible for azoospermia may be related to an obstruction or to Y-chromosome deletions. In addition, another 118 men were excluded because they had at least one of the following medical conditions that might alter semen quality: vasectomy, varicocele, orchiditis, epididymitis, vesiculitis, hernia repair complicated by testicular atrophy, injury of testis, or undescended testicle. A total of 2,009 men were included for final analysis.

*Questionnaires*. All of the study participants completed a face-to-face questionnaire under the guidance of trained investigators. The collected information included demographics, lifestyle habits, occupational exposures, medical characteristics, and routine water-use activities. Questions regarding routine water-use activities included the types of water source, the total volume of tap-water consumption per day (number multiplied by glass size), time spent showering/bathing per day (frequency multiplied by duration of bathing/showering), and the status of swimming (yes/no) in a chlorinated pool within the last 3 months. To allow for an accurate estimation of tap-water consumption, participants were provided a list of the volumes of commonly used containers (e.g., 150 mL for a plastic cup and 500 mL for a bottled-water container).

*Semen collection and analysis*. Before the collection of semen samples, participants were asked to report their period of abstinence from ejaculating. In a specialized semen collection room close to the semen laboratory, each participant was asked to masturbate into a sterile plastic specimen container. After liquefaction of the semen in a heating chamber (37°C) for ≤ 60 min, semen volume was measured with a serologic pipette. Sperm concentration, motility, and motion parameters were analyzed according to the World Health Organization (WHO 1999) guidelines using a Micro-cell slide and computer-aided semen analysis (WLJX 9000; Weili New Century Science & Tech Development Co. Ltd., Beijing, China). Sperm morphology was analyzed after staining using the modified Papanicolaou method recommended by the WHO (1999). Sperm count was calculated by multiplying the semen volume by the sperm concentration. Three conventional parameters for semen quality were reported: sperm concentration (million per milliliter), sperm count (million), and sperm motility (percent grades a and b motile sperm). Semen morphology parameters such as percent normal morphology and percent abnormal heads were also reported here. In addition, three principal parameters for the vigor and pattern of sperm motion were reported: straight-line velocity (VSL), curvilinear velocity (VCL), and linearity (LIN = VSL/VCL × 100). To reduce the variation in assessment of semen quality parameters, all semen samples were analyzed by the same two professional technicians. External quality controls were established by the semen laboratory according to the WHO (1999) guidelines.

*Urine collection and analysis*. We collected a single spot urine sample from each participant in the morning. After collection, all urine samples were packed into coolers with ice packs and sent to the laboratory for TCAA analysis to be performed within 6 months. TCAA concentrations in urine samples were analyzed in a blind fashion, according to the method described in detail in our previous study (Xie et al. 2011). Briefly, a 10-mL urine sample was extracted using methyl-*tert*-butyl-ether, which contained the internal standard 1,2-dipropyl bromide. The TCAA in the organic extraction was converted to its methyl ester by the addition of acidic methanol followed by heating at 50°C for 2 hr. Then, the acidic extraction was neutralized with a saturated solution of sodium bicarbonate. The target analyte was measured using gas chromatography (GC) coupled with an electron capture detector. One blank and two quality control samples were also analyzed along with each analysis run (30–40 samples). The limit of detection (LOD) for TCAA was 2.00 μg/L, and concentrations below the LOD were assigned the LOD divided by the square root of 2 for the analysis. Urinary creatinine was determined by the picric acid assay to adjust for the variation in urine diluteness, using commercial test kits purchased from Jiancheng Bioengineering Ltd. (Nanjing, China).

*Statistical analysis*. We performed statistical analysis using the Predictive Analytics Suite Workstation, version 18.0 (IBM Corporation, Armonk, NY, USA). We calculated descriptive statistics on the distributions of demographic characteristics, urinary TCAA concentrations, and semen quality parameters of study participants. To compare between-group differences in all continuous or categorical variables, parametric or nonparametric methods were appropriately used to test statistical significance.

We examined the relationship between urinary TCAA levels and semen quality using logistic regression models, in which study participants were dichotomized as either below or at/above WHO (1999) reference values for sperm concentration (20 million/mL), sperm motility (50% motile), and sperm count (40 million). Participants with all three parameters at or above the reference values were defined as the comparison group. The urinary TCAA levels were categorized into quartiles based on the distribution in the study population as a whole. We also used linear regression models to examine the relationship between urinary TCAA levels and the continuous measures of semen quality parameters. Because of the skewed distributions of sperm count and concentration, a natural-log transformation was applied to better achieve the normality assumption of the linear models.

Covariates were included in the multivariate models based on biological and statistical considerations. We included urinary creatinine as a separate independent variable in all models (Barr et al. 2005). We represented the crude models adjusted only for urinary creatinine. We used the change-in-effect estimate method to determine whether the potential confounders should be included in the multivariate models (Greenland 1989). Potential confounders were retained in the final models if including them changed the effect estimates for urinary TCAA and the outcomes [odds ratio (OR) or regression coefficient] by ≥ 10%. Most of the potential confounders [age, body mass index (BMI), ethnicity, abstinence time, smoking status, alcohol use, and education] did not meet the criterion. However, to facilitate comparisons with previously published studies (Fenster et al. 2003; Luben et al. 2007; Xie et al. 2011), here we report estimates from final models adjusted for age and urinary creatinine as continuous variables; education (≥ high school vs. < high school) as a dichotomous variable; and abstinence time (3–5 and > 5 vs. < 3 days), smoking status (current and former vs. never-smoker), and income (2,000–6,000 and > 6,000 vs. < 2,000 yuan/month) modeled using indicator variables. We defined statistical significance as a *p-*value < 0.05.

## Results

*Characteristics of study participants by semen parameters.* Table 1 lists the characteristics of study participants by semen parameter. Participants were primarily Han with a median age of 32 years. According to the WHO (1999) reference values, 253 men (12.6%) had sperm concentration below the reference (< 20 million/mL), 1,128 men (56.1%) had sperm motility below the reference (< 50% motile), and 206 men (10.2%) had sperm count below the reference (< 40 million). A total of 827 men (41.2%) had the three semen parameters at or above the reference values and were therefore defined as making up the comparison group. Alcohol use and income were each different between the comparison group and groups with sperm concentration, motility, and count below reference values (*p* < 0.05). Smoking and education were each different between the comparison group and groups with sperm concentration and motility below reference values (*p* < 0.05). However, we observed no significant differences in individual routine water-use activities among the semen quality groups except for total tap-water consumption, which differed between the comparison group and group with sperm concentration below reference value (*p* = 0.009).

**Table 1:** Distribution of characteristics [*n* (%) or mean ± SD] of study participants by semen parameters (*n* = 2,009^*a*^).

Characteristic	Comparison group^*b*^	Sperm concentration < 20 million/mL^*c*^	Sperm motility < 50% motile^*c*^	Sperm count < 40 million^*c*^
Observations (*n*)	827 (100)	253 (100)	1,128 (100)	206 (100)
Age (years)	31.9 ± 5.3	31.0 ± 5.5	32.2 ± 5.7	31.4 ± 6.0
BMI (kg/m^2^)	23.6 ± 3.8	23.2 ± 3.8	23.7 ± 4.2	23.2 ± 3.9
Ethnicity
Han	802 (97.0)	251 (99.2)	1,108 (98.4)	202 (98.5)
Other	25 (3.0)	2 (0.8)	18 (1.6)	3 (1.5)
Abstinence time (days)
< 3	80 (9.7)	29 (11.5)	85 (7.5)	26 (12.6)
3–5	530 (64.1)	153 (60.5)	684 (60.6)	136 (66.0)
> 5	217 (26.2)	71 (28.1)	359 (31.8)	44 (21.4)
Smoking status
Never-smoker	306 (37.2)	103 (40.9)	475 (42.3)	81 (39.5)
Former smoker	143 (17.4)	56 (22.2)	208 (18.5)	46 (22.4)
Current smoker	371 (31.4)	93 (36.9)	440 (39.2)	78 (38.0)
Alcohol use
Yes	680 (82.5)	186 (73.8)	842 (75.0)	151 (73.7)
No	144 (17.5)	66 (26.2)	280 (35.5)	54 (26.3)
Education
< High school	290 (35.5)	109 (43.4)	455 (40.6)	82 (40.2)
≥ High school	526 (64.5)	142 (56.6)	665 (59.4)	122 (59.8)
Income (RMB yuan/month)
< 2,000	217 (26.5)	89 (35.3)	346 (30.8)	76 (36.9)
2,000–6,000	477 (58.2)	142 (56.3)	666 (59.3)	112 (54.4)
≥ 6,000	125 (15.3)	21 (8.3)	111 (9.9)	18 (8.7)
Water source
Surface water	732 (88.5)	218 (86.2)	983 (87.1)	174 (84.5)
Groundwater	70 (8.5)	25 (9.9)	122 (10.8)	23 (11.2)
Both	25 (3.0)	10 (4.0)	23 (2.0)	9 (4.4)
Total tap-water consumption (mL/day)
< 1,000	553 (67.2)	192 (75.9)	796 (70.9)	147 (72.1)
≥ 1,000	270 (32.8)	61 (24.1)	326 (29.1)	57 (27.9)
Showering/bathing time (min/day)
< 10	408 (49.9)	121 (48.0)	564 (50.4)	97 (48.0)
≥ 10	409 (50.1)	131 (52.0)	554 (49.6)	105 (42.0)
Swimming in chlorinated pool
Yes	17 (2.1)	1 (0.4)	17 (1.5)	2 (1.0)
No	810 (97.9)	251 (99.6)	1,107 (98.5)	204 (99.0)
^***a***^Twenty-seven missing age, 7 missing BMI, 2 missing race, 9 missing smoking status and alcohol use, 19 missing education, 13 missing income, 12 missing total tap-water consumption, 21 missing showering/bathing time, and 4 missing swimming in chlorinated pool. ^***b***^Comparison group is study subjects with sperm concentration ≥ 20 million/mL, sperm count ≥ 40 million, and motility ≥ 50% motile. ^***c***^A subject may contribute data to more than one category.				

*Semen parameters and urinary TCAA concentrations.* Table 2 shows the distribution of semen parameters and urinary TCAA concentrations. The median sperm concentration, sperm motility, and sperm count were 50.83 million/mL, 46.67%, and 136.91 million, respectively. The mean sperm VSL, VCL, and LIN were 27.67 μm/sec, 43.69 μm/sec, and 63.62%, respectively. TCAA was detected in 98.6% of the urine samples (> LOD) from study participants. The mean (median) urinary TCAA concentration was 9.58 (7.97) μg/L (interquartile range, 6.01–10.96 μg/L).

**Table 2:** Distribution of semen parameters and urinary TCAA concentrations (*n* = 2,009).

Variable	Mean	Median	Percentile				Range
			10th	25th	75th	90th	
Semen quality
Sperm concentration (million/mL)	63.82	50.83	17.49	29.79	84.92	128.19	2.69–333.50
Sperm motility (% motile)	46.03	46.67	21.00	34.16	59.73	69.74	0.00–89.07
Sperm count (millions)	181.79	136.91	39.13	74.56	238.29	380.28	0.58–1238.73
Semen morphology
Normal morphology (%)	25.04	25.00	16.00	21.50	28.50	32.50	0.00–57.00
Abnormal head (%)	60.71	60.00	53.00	57.00	65.00	71.50	0.00–98.33
Sperm motion
VSL (μm/sec)	27.67	27.73	20.30	23.88	31.77	34.92	0.00–50.23
VCL (μm/sec)	43.69	43.75	32.14	37.52	50.48	55.82	0.00–73.90
LIN (%)	63.62	63.78	55.02	58.96	68.76	73.24	0.00–89.31
Urinary TCAA (μg/L)	9.58	7.97	4.56	6.01	10.96	16.15	LOD–81.74

*DBP exposure and semen quality*. Table 3 shows the associations of below-reference semen quality parameters with quartiles of urinary TCAA concentrations. Significant positive associations between TCAA above the lowest quartile were estimated for each outcome, although we did not observe monotonically increasing ORs with increasing TCAA quartiles. Adjusted ORs were slightly higher than crude ORs. Compared with those with the lowest urine levels of TCCA, men in the second and fourth quartiles had increased ORs of having below-reference sperm concentration (OR = 1.79; 95% CI: 1.19, 2.69 and OR = 1.51; 95% CI: 0.98, 2.31, respectively); men in the second and third quartiles had an increased OR of having below-reference sperm motility (OR = 1.46; 95% CI: 1.12, 1.90 and OR = 1.30; 95% CI: 1.00, 1.70, respectively); and men in the second quartile had an increased OR of having below-reference sperm count (OR = 1.62; 95% CI: 1.04, 2.55).

**Table 3:** ORs (95% CIs) for below-reference semen quality parameters (sperm concentration < 20 million/mL, sperm motility < 50% motile, sperm count < 40 million) with quartiles of urinary TCAA concentrations (*n* = 2,009).

TCAA quartile	*n*^*a*^	Sperm concentration		Sperm motility		Sperm count	
		*n*^*b*^	OR (95% CI)	*n*^*b*^	OR (95% CI)	*n*^*b*^	OR (95% CI)
Crude OR (μg/L)^*c*^
≤ 6.01	226	60	1.00 (Referent)	261	1.00 (Referent)	48	1.00 (Referent)
> 6.01–7.97	186	78	1.63 (1.10, 2.41)	308	1.45 (1.12, 1.88)	59	1.53 (1.00, 2.36)
> 7.97–10.96	201	47	0.94 (0.61, 1.45)	290	1.28 (0.99, 1.66)	49	1.22 (0.77, 1.91)
> 10.96	214	68	1.29 (0.86, 1.95)	269	1.12 (0.86, 1.46)	50	1.18 (0.75, 1.86)
Adjusted OR (μg/L)^*d*^
≤ 6.01	226	60	1.00 (Referent)	261	1.00 (Referent)	48	1.00 (Referent)
> 6.01–7.97	186	78	1.79 (1.19, 2.69)	308	1.46 (1.12, 1.90)	59	1.62 (1.04, 2.55)
> 7.97–10.96	201	47	0.96 (0.61, 1.50)	290	1.30 (1.00, 1.70)	49	1.28 (0.80, 2.04)
> 10.96	214	68	1.51 (0.98, 2.31)^*e*^	269	1.19 (0.90, 1.56)	50	1.41 (0.88, 2.26)
^***a***^Number of subjects in each exposure quartile with sperm concentration ≥ 20 million/mL, sperm count ≥ 40 million and motility ≥ 50% motile. ^***b***^Number of subjects in each exposure quartile with below-reference semen quality parameters. ^***c***^Adjusted for urinary crea­tinine (continuous). ^***d***^Adjusted for urinary crea­tinine and age (continuous), education (≥ high school vs. < high school), abstinence time (3–5 and > 5 vs. < 3 days), income (2,000–6,000 and > 6,000 vs. < 2,000 yuan/month), and smoking status (current and former vs. never-smoker). ^***e***^*p*-Value = 0.059.							

For the continuous outcomes, adjusted estimates were also similar to crude estimates (Table 4). In adjusted models, compared with those in the first quartile of urinary TCAA, men in the second and fourth quartiles had significant proportional decreases in sperm concentration of 0.17 (95% CI: –0.30, –0.06) and 0.14 (95% CI: –0.26, –0.03), respectively; men in the second, third, and fourth quartiles had significant decreases in sperm motility of 4.22% (95% CI: –6.55, –1.88), 2.81% (95% CI: –5.18, –0.45), and 2.86% (95% CI: –5.28, –0.45), respectively; men in the second quartile had a significant proportional decrease in sperm count of 0.13 (95% CI: –0.27, –0.01); and men in the fourth quartile had a proportional decrease in sperm count of 0.12 (95% CI: –0.26, 0.01), although it was not statistically significant. In addition, we found that men in the second quartile had a significant decrease in percent normal morphology of 0.89% (95% CI: –1.76%, –0.17%) compared with those in the first quartile of urinary TCAA (Table 5). However, we found that urinary TCAA levels were not significantly associated with sperm VSL, VCL, or LIN (Table 6).

**Table 4:** Regression coefficients (βs) (95% CIs) for semen quality parameters associated with quartiles of urinary TCAA concentrations (*n* = 2,009).

TCAA quartile	Sperm concentration (proportional difference)^*a*^	Sperm motility (percent motile)	Sperm count (proportional difference)^*a*^
Crude (μg/L)^*b*^
≤ 6.01	0 (Referent)	0 (Referent)	0 (Referent)
> 6.01–7.97	–0.16 (–0.28, –0.06)	–4.24 (–6.53, –1.94)	–0.12 (–0.26, 0.00)
> 7.97–10.96	–0.04 (–0.15, 0.05)	–2.46 (–4.79, –0.12)	–0.04 (–0.17, 0.07)
> 10.96	–0.12 (–0.23, –0.01)	–2.31 (–4.69, 0.06)	–0.09 (–0.23, 0.02)
Adjusted (μg/L)^*c*^
≤ 6.01	0 (Referent)	0 (Referent)	0 (Referent)
> 6.01–7.97	–0.17 (–0.30, –0.06)	–4.22 (–6.55, –1.88)	–0.13 (–0.27, –0.01)
> 7.97–10.96	–0.06 (–0.17, 0.04)	–2.81 (–5.18, –0.45)	–0.06 (–0.20, 0.05)
> 10.96	–0.14 (–0.26, –0.03)	–2.86 (–5.28, –0.45)	–0.12 (–0.26, 0.01)
^***a***^Sperm concentration and count was natural-log transformed. ^***b***^Adjusted for urinary crea­tinine (continuous). ^***c***^Adjusted for age, urinary crea­tinine (continuous), education (≥ high school vs. < high school), abstinence time (3–5 and > 5 vs. < 3 days), income (2,000–6,000 and > 6,000 vs. < 2,000 yuan/month), and smoking status (current and former vs. never-smoker).			

**Table 5:** Regression coefficients (βs) (95% CIs) for semen morphology parameters associated with quartiles of urinary TCAA concentrations (*n* = 2,009).

TCAA quartile	Percent normal morphology (%)	Percent abnormal head (%)
Crude (μg/L)^*a*^
≤ 6.01	0 (Referent)	0 (Referent)
> 6.01–7.97	–0.80 (–1.65, 0.05)	0.34 (–0.64, 1.33)
> 7.97–10.96	–0.12 (–0.98, 0.75)	–0.46 (–1.47, 0.54)
> 10.96	0.78 (–0.10, 1.66)	–2.05 (–3.07, –1.03)
Adjusted (μg/L)^*b*^
≤ 6.01	0 (Referent)	0 (Referent)
> 6.01–7.97	–0.89 (–1.76, –0.17)	0.49 (–0.52, 1.50)
> 7.97–10.96	–0.20 (–1.09, 0.68)	–0.45 (–1.47, 0.58)
> 10.96	0.74 (–0.16, 1.65)	–2.04 (–3.08, –0.99)
^***a***^Adjusted for urinary crea­tinine (continuous). ^***b***^Adjusted for age, urinary crea­tinine (continuous), education (≥ high school vs. < high school), abstinence time (3–5 and > 5 vs. < 3 days), income (2,000–6,000 and > 6,000 vs. < 2,000 yuan/month), and smoking status (current and former vs. never-smoker).		

**Table 6:** Regression coefficients (βs) (95% CIs) for sperm motion parameters associated with quartiles of urinary TCAA concentrations (*n* = 2,009).

TCAA quartile	VSL (μm/sec)	VCL (μm/sec)	LIN (%)
Crude (μg/L)^*a*^
≤ 6.01	0 (Referent)	0 (Referent)	0 (Referent)
> 6.01–7.97	–0.33 (–1.09, 0.43)	–0.38 (–1.59, 0.84)	–0.32 (–1.39, 0.74)
> 7.97–10.96	0.09 (–0.69, 0.86)	0.23 (–1.00, 1.47)	–0.68 (–1.77, 0.40)
> 10.96	0.23 (–0.55, 1.02)	0.32 (–0.93, 1.58)	–0.56 (–1.66, 0.54)
Adjusted (μg/L)^*b*^
≤ 6.01	0 (Referent)	0 (Referent)	0 (Referent)
> 6.01–7.97	–0.37 (–1.14, 0.40)	–0.41 (–1.65, 0.83)	–0.35 (–1.45, 0.74)
> 7.97–10.96	–0.04 (–0.83, 0.74)	0.06 (–1.20, 1.31)	–0.71 (–1.82, 0.40)
> 10.96	–0.01 (–0.80, 0.80)	0.06 (–1.22, 1.34)	–0.71 (–1.84, 0.43)
^***a***^Adjusted for urinary crea­tinine (continuous). ^***b***^Adjusted for age, urinary crea­tinine (continuous), education (≥ high school vs. < high school), abstinence time (3–5 and > 5 vs. < 3 days), income (2,000–6,000 and > 6,000 vs. < 2,000 yuan/month), and smoking status (current and former vs. never-smoker).			

## Discussion

We conducted the first large-scale cross-sectional study to examine the association between exposure to drinking-water DBPs and semen quality in a Chinese population, using urinary TCAA as a biomarker. In general, urinary TCAA levels above the lowest quartile were associated with lower semen quality, based on multivariable models of dichotomous outcomes (resulting in positive ORs for semen quality parameters below vs. above the reference level) and multivariable linear regression models (resulting in negative linear regression model coefficients for semen quality parameters modeled as continuous variables), although the magnitude of associations did not increase monotonically with increasing quartiles of exposure, and not all associations were above the null or statistically significant. In addition, we observed that men in the second quartile of urinary TCAA were significantly associated with decreased percent normal morphology compared with those in the first quartile. Our findings indicated associations between urinary TCAA levels above the lowest quartile and multiple indicators of lower semen quality, consistent with previous toxicological studies (Klinefelter et al. 1995, 2002, 2004; Linder et al. 1994a, 1994b, 1995, 1997a).

To date, the relationship between exposure to drinking-water DBPs and semen quality has been examined in several epidemiological studies with inconsistent results. Previous studies used DBP concentrations in drinking water as surrogates of exposure. Fenster et al. (2003) reported that exposure to THMs, the most abundant class of DBPs, was not associated with decreased semen quality, with the exception of bromodichloromethane, which was inversely associated with sperm linearity in healthy men. Luben et al. (2007) also found no association between exposure to DBPs at levels approaching regulated limits and decreased semen quality in presumed fertile men. Our results were not consistent with these findings. This discrepancy might be attributed to the different study populations and the different exposure assessments (including the potentially different exposure levels). In recent studies, we used biomarkers of DBP exposures, including THMs in whole blood and TCAA in urine, to improve our assessment of exposure (Xie et al. 2011; Zeng et al. 2013). We found evidence suggestive of an association between elevated urinary TCAA levels and decreased sperm motility. We also found that baseline blood THM concentrations were associated with decreased sperm count and concentration. However, limited sample sizes made it difficult to comprehensively understand the effect of exposure to drinking-water DBPs on semen quality. To our knowledge, no large-scale epidemiological study has been published to date.

Because of the complexity of exposure to hundreds of different chemicals and the multiple routes contributing to exposure, exposure assessment is one of the main limitations in epidemiological studies of drinking-water DBPs and reproductive health (Savitz 2012). However, biomarkers offer great promise for enhancing the assessment of exposure (Esteban and Castaño 2009). Thus far, biomarkers of DBP exposure, including THMs in blood or expired air and TCAA in urine, have been developed (Blount et al. 2006; Froese et al. 2002; Kim et al. 1999; Rivera-Núñez et al. 2012). THMs are volatile compounds that are rapidly metabolized in the body and exhaled after ingestion, inhalation, and dermal contact, limiting the accuracy of THMs measured in blood and expired air and making them difficult to use as measures of chronic DBP exposure. Furthermore, the collection of blood and expired air samples is invasive and limits their use in large-scale epidemiological studies. However, TCAA has a longer half-life of elimination in the body, and its excretion in urine has been shown to significantly correlate with its ingestion via drinking water (Zhang et al. 2009a). Moreover, the collection of urine samples is noninvasive and thus feasible for large-scale epidemiological studies. Previous studies have reported that urinary TCAA could be a valid biomarker of DBP ingestion (e.g., HAAs) through chlorinated drinking water (Calafat et al. 2003; Froese et al. 2002; Kim et al. 1999; Zhang et al. 2009b). A recent study has found that urinary TCAA concentrations were significantly associated with THM ingestion (total THMs and all individual THMs except bromoform) (Costet et al. 2012).

Because potential effects of exposure to DBPs in drinking water on semen quality would reflect exposure over a 3-month period (90 days corresponds to the period of spermatogenesis) and exposure to DBPs is likely to vary over time (both within and between days) as a result of changes in routine water-use activities, the relevance of measuring urinary TCAA concentration in a single spot sample has been debated. Several studies have reported substantial inter- and intraindividual variability in urinary TCAA concentrations (Froese et al. 2002; Zhang et al. 2009a). A recent study also reported that 2-day urine sampling resulted in a higher correlation between estimated TCAA ingestion and urinary TCAA concentration than 1-day sampling (Zhang et al. 2009a). However, a 2-day sampling strategy is not practical in large-scale epidemiological studies. Thus, a single spot urine sample as used in the present study may not be a reliable measure of DBP exposure over time.

Although the urinary TCAA analysis used in the epidemiological studies could improve the assessment of exposure, it is also notable that the biomarker may overestimate or underestimate actual exposure levels. Exposure to drinking-water DBPs is not a unique source of urinary TCAA, and several other chemicals, such as TCE, TRI and PERC, can also be metabolized into urinary TCAA (ATSDR, 1995, 1996, 1997). Although we excluded from our present study participants who reported occupational exposure to potential sources of these chemicals, household or workplace exposure to these chemicals cannot be ruled out. Calafat et al. (2003) reported that urinary TCAA concentrations in the general population may be, at least in part, associated with exposure to TCE and TRI. Although urinary TCAA has been shown to reflect DBP exposure through the ingestion of chlorinated drinking water, humans also are exposed to DBPs in chlorinated water through inhalation and dermal absorption, especially the volatile DBPs (e.g., THMs). In addition, urinary TCAA has not been evaluated as a marker of exposure to brominated HAAs, which may be more potent testicular toxicants than dichloro analogues based on toxicological studies of rats (Linder et al. 1994b, 1995). Therefore, the extent to which our results might apply to DBP exposure in general is unknown.

In our study, we addressed several limitations. First, we performed a cross-sectional study in which exposure was estimated based on a single spot urine sample that may not have reflected exposure during the etiologically relevant time window for effects on semen quality. In addition, some exposure misclassification due to the substantial inter- and intraindividual variability in urinary TCAA is likely. Such misclassification might obscure a monotonic exposure–response relationship, but a bias away from the null for associations with individual quartiles of exposure also cannot be ruled out. Future studies should longitudinally examine the association between exposure to drinking-water DBPs and semen quality. Second, our study was a hospital-based study in an infertility clinic. This study design facilitated participation, but the resulting study population might not be representative of the general population. Future studies in the general population also need to confirm associations between DBP exposure and semen quality parameters. Finally, we only collected a single semen sample from each participant for measurement of semen quality. Although two recent studies have found that the within-subject ﬂuctuations of semen quality are small (Francavilla et al. 2007; Stokes-Riner et al. 2007), exposure over the previous several weeks may not capture the etiologically relevant time window for effects on semen quality.

## Conclusions

In summary, our large-scale study provided some evidence that exposure to drinking-water DBPs may contribute to decreased semen quality in humans. Our findings were consistent with previous toxicological data. However, the potential effects of exposure to drinking-water DBPs on human semen quality warrants still further study in the general population.
